# Supplementary material for: A Collagen Basketweave from the Giant Squid Mantle as a Robust Scaffold for Tissue Engineering
Source: Mar Drugs. 2021 Nov 29;19(12):679. doi: 10.3390/md19120679 (PMC8706038; doi:10.3390/md19120679)
Supplement: Supplementary file 1 [file marinedrugs-19-00679-s001.zip › marinedrugs-1491397-supplementary.pdf]

Supplementary Materials

Table S1. Band positions for the GSCM, collagen Type I and II.

Sample	Collagen membrane of squid	Collagen type II	Collagen type I
Wavenumber, cm^{-1}	-	-	3687
	-	-	3649
	-	-	3617
	3288	3299	3301
	3073	3073	3078
	2958	2963	3004
	2927	2931	2921
	2878	2876	-
	2854	2847	2851
	1740	1732	1745
	1630	1630	1632
	1540	1542	1547
	1451	1451	1454
	1382	1398	1401
	1336	1337	1379
	1314	1314	1341
	1278	1278	1278
	1236	1237	1238
	1204	1203	1203
	1158	1160	1161
	1119	1124	1114
	1080	1080	1082
	1060	1061	1059
	1030	1030	1031
	972	972	1008
	939	946	941
	918	919	915
	876	876	871

A preliminary in vivo study of the GSCM biosafety

$scCO_2$ extraction of the GSCM

The treatment of samples in the supercritical carbon dioxide medium was performed in a high-pressure reactor of a 60 cm^3 volume, equipped with a heater, magnetic stirrer, thermocouple and a manometer. The reactor was preliminarily cooled to ~ 0 ºC, the GSCM samples were placed inside, and the reactor was filled with CO_2 (99.8 % purity by GOST 8050-85, NII KM Company) at room temperature (25 ºC), and the gas was compressed up to 20-22 MPa. Then, the reactor was gradually heated to 37 ºC and pressurized to 25 MPa. The samples were kept for 3 hours in the reactor with the medium stirred. Then, slowly (during 30 min) the reactor was cooled to room temperature, and CO_2 was released through the reactor outlet during 20 min.

Heterotopic implantation

The tissue response was assessed by heterotopic subcutaneous implantation of GSCM to male Wistar rats with the weight of 230 ± 20 g (n=12). All the animal procedures were conducted in accordance with the European convention (Strasbourg, 1986) concerning a humane animal treatment, after the approval of the Local Ethical Committee of the Sechenov University (Committee Meeting No. 67 of August 25, 2021, Moscow, Russia). The rats were kept in the standard vivarium conditions with 2-3 animals per cage, provided with a complex granulated feed and a constant access to water. The animals were acclimatized in the room during 14 days. In order to exclude somatic pathology, a general veterinary examination was performed one week before the surgical intervention. The rats were anesthetized with an intramuscular injection of a mixture of zoletil (20 mg/kg) and xylazine (5 mg/kg). From the moment of inducing the anesthesia to the moment of getting out the anesthesia the animals were kept on a heated table with a sustained temperature of 37°C. In a preliminarily trimmed interscapular region, a 2 cm-long skin incision was performed with a scalpel in aseptic conditions. The subcutaneous fat was dissected in a blunt manner in the directions towards the right and left scapula, with the creation of two 2 cm-deep pockets. The formed cavities were rinsed with an aqueous solution of chlrohexidine, and sterile samples with the sizes of 1.0x0.5 cm were placed inside: the GSCM treated with scCO2 on the right side, the intact GSCM on the left side. The samples were sutured to the tissues with 3-4 interrupted sutures with prolene 4.0. Following the adequate hemostasis, the surgical wound was thoroughly rinsed with antiseptic solutions, sutured tightly involving the muscle tissue and treated with Iodopyron. A general status of animals, body temperature, skin appearance above the implantation region were assessed in the dynamics. To prevent purulent-septic complications, the surgical wound debridement and injections of 2.5% baytril, an antibacterial drug, in the concentration of 5 mg/kg were performed for 7 days. To relief the pain syndrome, a ketonal solution at the dose of 1 mg/kg was administered subcutaneously. The animals were sacrificed in 14 days 14 (n=6) and 30 days (n=6) after the implantation by carbon dioxide inhalation. The two implants with the capsules formed around involving the muscle tissue with the adjacent skin flaps, of a 0.5 cm thickness, were separated. The tissue fragments with the experimental sample were rinsed with the saline and fixed in 10% formalin.

In the early post-surgical period, all the animals were active, with an intact general status, the body temperature was in the normal range (38.5-39.5 °C), the food behavior was normal. In 10

days, the wound was healed by primary intention, no reactive changes were observed in the surrounding tissues.

Morphological and optical characteristics of the implanted intact GSCM and GSCM after scCO2-treatment

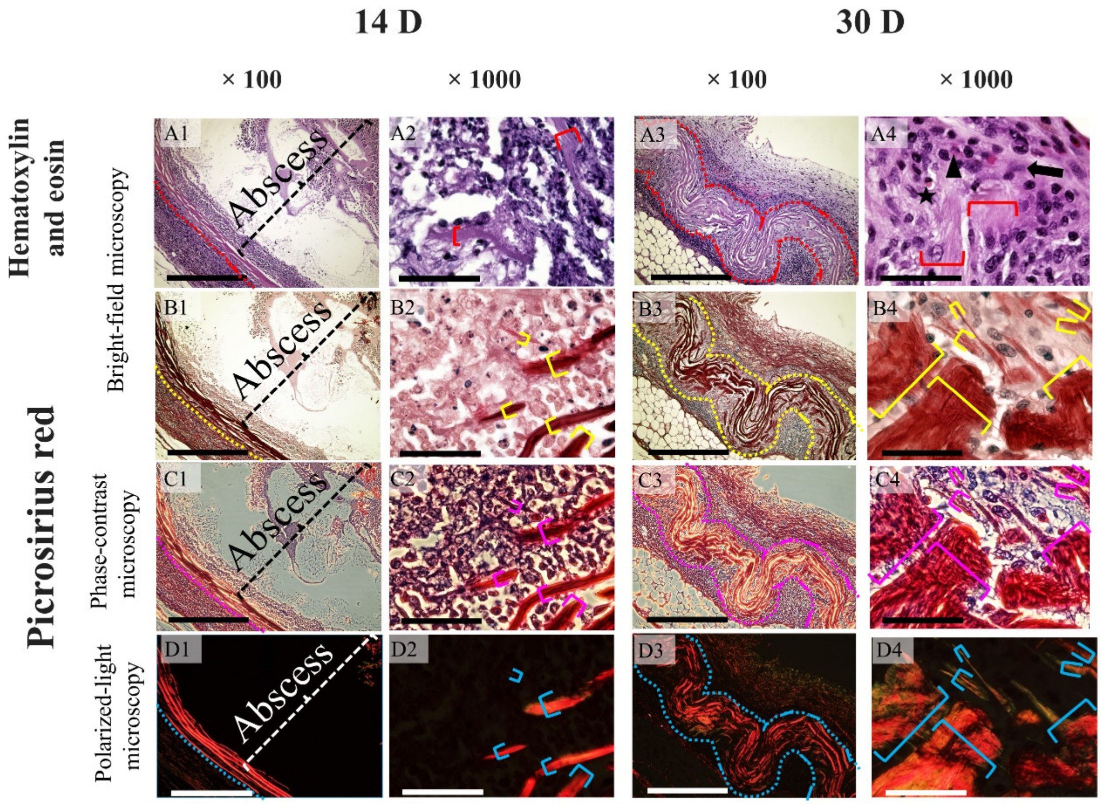

Figure S1. Morphological and optical characteristics of the implanted intact GSCM. A1 – on day 14, a significant inflammatory infiltration of the GSCM material and surrounding tissues (up to purulent fusion with the formation of abscesses) was noted in most of the animals. In the intact regions, the GSCM thickening up to 200-300 μm due to the material unweaving and swelling was seen (the border is marked with a dashed line), as well as the formation of a capsule from the maturing granulation tissue; A2 – at a higher magnification, among the tissue debris, microbial colonies and the remnants of the lysed "laminae" were observed (marked with square brackets) with the thickness of up to 10 μm; A3, A4 – on day 30, the inflammatory infiltration of the GSCM and adjacent tissues was reduced, the residual signs of mixed-cell inflammation and insignificant compaction of "laminae" with the increased distance between them being observed in the intact regions, in the regions of residual inflammation small blood vessels (*), fibroblasts (arrow) and macrophages (arrowtip) were visualized between and inside the remnants of the lysed "laminae"; B1–B4 - on days 14 and 30, collagen fibers in the intact "laminae" had bright-red coloration when stained with picrosirius red, while even on day 30 after the implantation, the capsule around the GSCM contained a small number of collagen fibers; C1, C2 – in phase contrast microscopy images, a crispier structure of loosened "laminae" within the intact GSCM regions was noted; C3, C4 – a compact packing of collagen fibers within the intact "laminae" with the presence of regions of thinned "laminae" and single thin collagen fibers between them (signs of partial resorption); D1 – D4 – in polarized light microscopy images, as compared to the other visualization methods, the intact "laminae" were the crispiest due to the bright glow of collagen fibers within them: the "laminae" produced a uniform yellow-orange and orange-red glow, with the appearance of weakly glowing yellow-green regions on day 30, while the glow of collagen fibers were absent in the adjacent capsule even on day 30. Magnification ×100, scale

bar - 500 µm; magnification ×1000, scale bar - 50 µm.

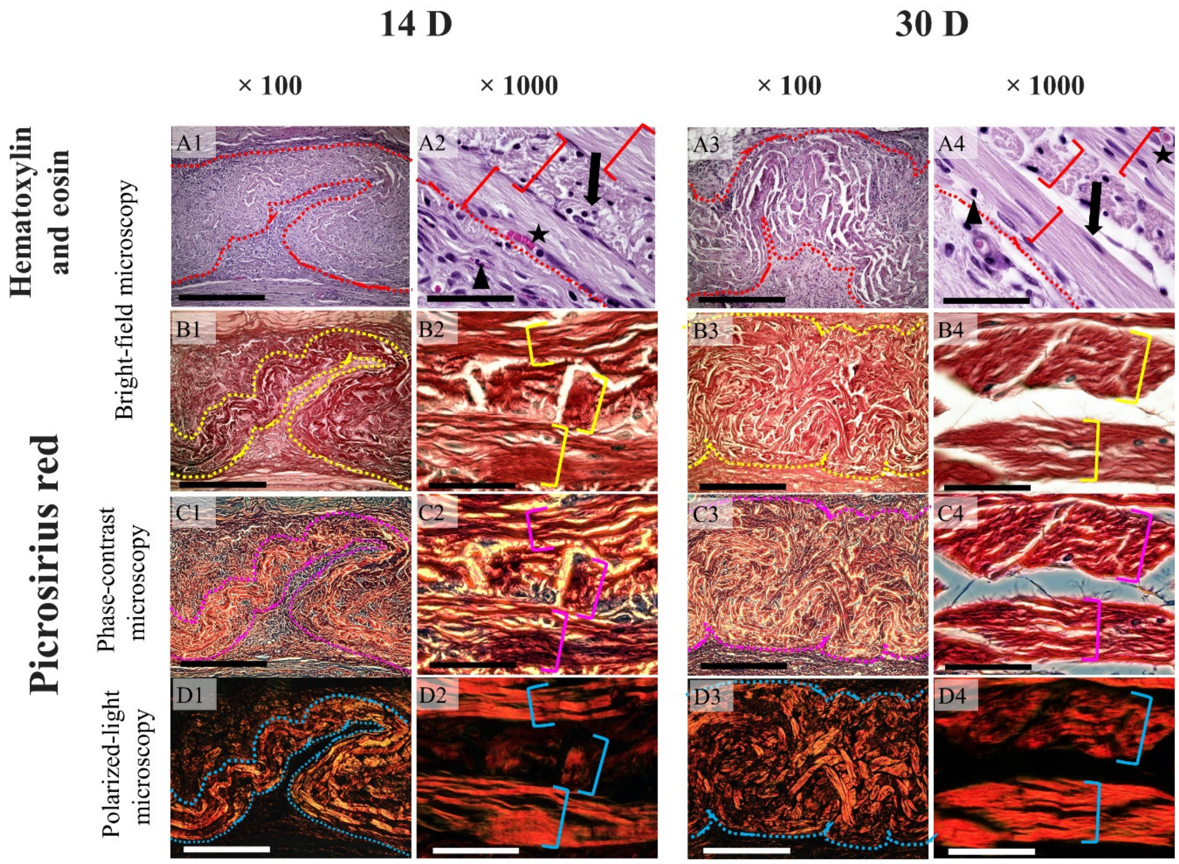

Figure S2. Morphological and optical characteristics of the implanted GSCM after the scCO2-treatment. A1 – on day 14, significant GSCM expansion was noted – up to ~ 500 µm (the border is marked with a dashed line), and a capsule from the maturing granulation tissue formed around the implant; A2 – at a higher magnification, swollen and unwoven “laminae” are seen (marked with square brackets) with the thickness of ~ 30-40 µm, small blood vessels (*), fibroblasts (arrow), macrophages (arrowtip) were visualized between and inside them, as well as in the adjacent capsule; A3 – on day 30, insignificant compaction of the “laminae” was noted with the increasing distance between them; A4 – at a higher magnification, it is seen that, in comparison with the previous time point, blood vessels are also noted inside the GSCM and the adjacent capsule, however, the number of fibroblasts and macrophages is lower, and the signs of the scaffold material resorption are almost absent; B1 – on day 14, the GSCM “laminae” and collagen fibers of the surrounding capsule had bright-red coloration when stained with picrosirius red; B2 – at a higher magnification, pronounced unweaving of collagen fibers within the “laminae” was seen; B3, B4 – on day 30, compaction of collagen fibers within the “laminae” was noted, with the red coloration remaining unchanged; C1, C2 – in phase contrast microscopy images, a crispier fibrous structure of loosened “laminae” was noted; C3, C4 – compaction of collagen fibers within the “laminae” with the presence of single thin collagen fibers between them; D1 – D4 – in polarized light microscopy images, as compared to the other visualization methods, the “laminae” within the GSCM were the crispiest due to the brighter glow, as compared to the adjacent capsule: collagen fibers within the “laminae” produced a uniform yellow-orange and orange-red glow, while collagen fibers in the adjacent capsule was characterized by non-uniform yellow-green, yellow-orange and orange-red glow. Magnification ×100, scale bar - 500 µm; magnification ×1000, scale bar - 50 µm.

The histology study revealed dramatic expansion of the GSCM (up to 4-10 times) on day 14 post-implantation, associated with both the increase in the distance between the “laminae” and

unweaving of collagen fibers within the “laminae” themselves (Figures S1 & S2). At the same time, the implantation of the intact GSCM was accompanied by a pronounced inflammatory reaction with purulent fusion of the material and surrounding tissues up to formation of large abscesses (see Figure S1: A1 & A2, B1 & B2, C1 & C2, D1 & D2). In contrast, the GSCM implantation after the $scCO_2$ treatment was accompanied by only insignificant inflammatory infiltration, numerous fibroblasts were noted in the superficial and deep layers of the material, and a maturing capsule was seen on the implant periphery (see Figure S2: A1 & A2, B1 & B2, C1 & C2, D1 & D2).

On day 30, in both groups the inflammatory infiltration around the GSCM samples was reduced, that was observed most drastically in the intact GSCM group. However, moderate mixed-cellular inflammatory infiltration was still seen on the periphery and inside the control samples (see Figure S1: A3 & A4, B3 & B4, C3 & C4, D3 & D4, Figure S2: A3 & A4, B3 & B4, C3 & C4, D3 & D4). In both groups, moderate compaction of collagen fibers within the “laminae” was also noted, with the increased distance between them. The signs of the GSCM material lysis characteristic of the collagenase treatment (Figure 4) were absent in both groups at this time point. However, in the intact GSCM groups, the processes of the “laminae” resorption continued due to the continuing inflammatory process.
